# Array CGH improves detection of mutations in the *GALC* gene associated with Krabbe disease

**DOI:** 10.1186/1750-1172-7-38

**Published:** 2012-06-15

**Authors:** Alice K Tanner, Ephrem L H Chin, Patricia K Duffner, Madhuri Hegde

**Affiliations:** 1 Emory Genetics Laboratory, Department of Human Genetics, Emory University, Atlanta, GA, USA; 2 Hunter James Kelly Research Institute, Department of Neurology, School of Medicine, State University of New York at Buffalo, Buffalo, NY, USA

**Keywords:** Krabbe disease, GALC, Deletion, Duplication, Array CGH

## Abstract

**Background:**

Krabbe disease is an autosomal recessive lysosomal storage disorder caused by mutations in the *GALC* gene. The most common mutation in the Caucasian population is a 30-kb deletion of exons 11 through 17. There are few other reports of intragenic *GALC* deletions or duplications, due in part to difficulties detecting them.

**Methods and results:**

We used gene-targeted array comparative genomic hybridization (CGH) to analyze the *GALC* gene in individuals with Krabbe disease in whom sequence analysis with 30-kb deletion analysis identified only one mutation. In our sample of 33 cases, traditional approaches failed to identify two pathogenic mutations in five (15.2%) individuals with confirmed Krabbe disease. The addition of array CGH deletion/duplication analysis to the genetic testing strategy led to the identification of a second pathogenic mutation in three (9.1%) of these five individuals. In all three cases, the deletion or duplication identified through array CGH was a novel *GALC* mutation, including the only reported duplication in the *GALC* gene, which would have been missed by traditional testing methodologies. We report these three cases in detail. The second mutation remains unknown in the remaining two individuals (6.1%), despite our full battery of testing.

**Conclusions:**

Analysis of the *GALC* gene using array CGH deletion/duplication testing increased the two-mutation detection rate from 84.8% to 93.9% in affected individuals. Better mutation detection rates are important for improving molecular diagnosis of Krabbe disease, as well as for providing prenatal and carrier testing in family members.

## Background

Krabbe disease, also called globoid cell leukodystrophy, is an autosomal recessive lysosomal storage disorder involving progressive damage to the white matter of the central and peripheral nervous systems (reviewed in [1]). The disease is caused by deficiency of the enzyme galactocerebrosidase (GALC), which leads to an inability to degrade galactolipids found mainly in the myelin sheath [2]. Symptoms include spasticity, irritability, and developmental delay and regression, which progress to a severe decerebrate condition with no voluntary movements [3]. While the age of onset and progression of the disease is variable, 85-90% of affected individuals develop symptoms in the first six months of life, with a median survival of 17 months [1,4]. Death is often due to respiratory infections or cerebral hyperpyrexia. Late infantile- and juvenile- onset forms with longer survival periods are also seen, and adults may present with weakness, loss of manual dexterity, and paresthesia in their extremities [1]. The prevalence of Krabbe disease is approximately one in 100,000 in the US and Europe [5] with higher frequencies in the Druze and Muslim Arabs in Israel [6].

Mutations in the *GALC* gene (14q31) cause Krabbe disease [7], and numerous nonsense, missense, small insertion, and small deletion mutations spanning the entire length of the *GALC* gene have been described [3]. The most common mutation, consisting of approximately 40% of alleles from affected individuals with European ancestry and 35% of alleles from those with Mexican ancestry, is a 30-kb deletion beginning in intron 10 and extending nine kb beyond the polyadenylation signal [3,8,9]. The 30-kb deletion mutation results in the classic infantile form of the disease in the homozygous state or when *in trans* (on opposite chromosomes) with another mutation associated with severe disease [3].

There are few reports of any other large deletions or large duplications encompassing the *GALC* gene. The techniques currently available for detecting single- and multi-exon deletions and duplications include multiplex PCR, quantitative PCR, Southern blotting, multiplex ligation-dependent probe amplification (MLPA), detection of virtually all mutations-SSCP (DOVAM-S), and single condition amplification/internal primer sequencing (SCAIP). Due to the difficulty and complexity of test development to routinely and reliably detect dosage differences, clinical laboratories have not offered this testing, and therefore data on the presence of these types of mutations are lacking.

To further explore the deletion/duplication mutation spectrum of the *GALC* gene, we have developed a comprehensive mutation detection strategy which begins with sequence analysis of the *GALC* gene in combination with mutation-specific testing for the 30-kb deletion. If two mutations are not found using this approach, despite an established biochemical diagnosis for the patient, this testing is followed by targeted array comparative genomic hybridization (CGH) to look for copy number changes within the *GALC* gene. Here we present mutation identification statistics for *GALC* analysis at Emory Genetics Laboratory (EGL); we also identify two novel *GALC* deletions and describe the only large *GALC* duplication reported in an individual with Krabbe disease.

## Results

Emory Genetics Laboratory (EGL) started offering array CGH-based deletion/duplication testing for the *GALC* gene in January of 2008. From January of 2008 through August of 2011, approximately 100 samples were submitted to EGL for full *GALC* gene analysis. There were 33 cases in which we performed complete *GALC* gene analysis (sequence analysis with 30-kb deletion analysis followed by array CGH deletion/duplication analysis, if necessary). These cases included samples from individuals reported to be enzymatically or clinically affected by Krabbe disease, and also paired parental samples from cases in which the affected individual was deceased. The remainder of the samples were not informative for our study and included samples for which no clinical information was submitted, samples sent for carrier testing in individuals with a family history of Krabbe disease in which the familial mutation was not known, and samples sent for comprehensive carrier testing from individuals with partners known to be carriers for Krabbe disease. Analysis began with sequencing of the 17 exons and flanking intronic regions of the *GALC* gene, along with allele-specific PCR analysis for the common 30-kb deletion. If a mutation(s) was not found, the ordering physician had the option of reflex testing with array CGH to detect single and multiple exon deletions and duplications.

Results of *GALC* analysis for the 33 cases of confirmed Krabbe disease are given in table 1. In 28 of the 33 cases (84.8%), both mutations were identified via sequence analysis in combination with analysis for the 30-kb deletion. Sixteen of these cases had two mutations identified through sequence analysis, eight had one mutation identified through sequence analysis and one copy of the 30-kb deletion, and four had a homozygous 30-kb deletion. In an additional three of the 33 cases (9.1%), sequence analysis with analysis of the 30-kb deletion identified one mutation, while reflex deletion/duplication testing identified a second mutation; these cases are discussed in more detail below as cases 1-3. The overall detection rate for two mutations using a combination of sequence analysis and array-CGH deletion/duplication testing was therefore 31/33 (93.9%).

**Table 1:** ***GALC*Mutations Identified in 33 Biochemically Confirmed Krabbe Disease Patients at Emory Genetics Laboratory**

**Mutation Category**	**# of Patients**	**% of Patients**
Two point mutations	16	16/33 = 48.5%
One point mutation and one 30-kb deletion	8	8/33 = 24.2%
Two 30-kb deletions	4	4/33 = 12.1%
One known mutation and one large deletion or duplication	3	3/33 = 9.1%
One known mutation and negative array CGH	2	2/33 = 6.1%

In two of the 33 cases (6.1%), comprehensive *GALC* mutation analysis, including sequence analysis, 30-kb deletion analysis, and deletion/duplication analysis, failed to identify two known mutations. When only one mutation is identified in individuals with a biochemical diagnosis of a recessive disease, it is EGL’s customary practice to design a second set of PCR primers for the entire gene involved. Resequencing of the *GALC* gene with an alternative primer set also failed to identify a second mutation in these individuals, reducing the probability of allele drop out. In one of the two cases, the proband was deceased but reportedly had clinical features consistent with Krabbe disease and an enzymatic diagnosis of Krabbe disease from another laboratory (laboratory reports of the enzyme analysis were not provided to EGL). Sequence analysis with 30-kb deletion analysis of the parents identified one copy of the 30-kb deletion in the mother, but was negative in the father. Reflex deletion/duplication testing in the father was also negative. In the second case, the proband had a biochemical diagnosis of Krabbe disease from another laboratory; no other information on the proband was provided. Sequence analysis with 30-kb deletion analysis identified one missense mutation and one intronic variant of unknown clinical significance (c.909-10A > G; IVS8-10A > G) in the affected individual. Reflex deletion/duplication testing was negative. Testing the parents of the second individual was recommended to aid in interpretation of the unknown variant, but has not been ordered at EGL to date.

### Case 1

**Figure 1:**
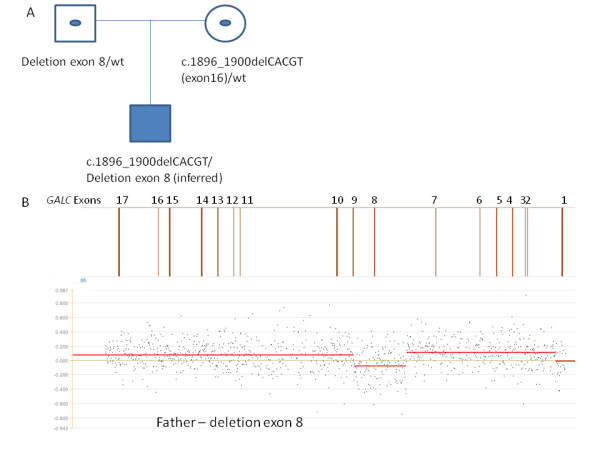
**Case 1. A. Pedigree of the proband and parents.** A filled symbol indicates the affected individual; symbols with a dot in the middle indicate carriers. *GALC* genotypes are given for each individual under the respective symbol. Sequence analysis with 30-kb deletion analysis identified one copy of a five-bp deletion in exon 16 in the proband and his mother. A second mutation was not identified. Array CGH analysis was performed on the proband’s father to identify the second mutation. **B.***GALC* array CGH results for the father. A diagram of the *GALC* gene is given above the results with exon numbers indicated. The father carries a deletion of exon 8 of the *GALC* gene. (The presence of the deletion of exon 8 in the proband is inferred, as the proband passed away before deletion/duplication analysis was performed).

Case 1 was an eight-month-old Caucasian male reported to have deficient GALC enzyme activity. Sequence analysis with 30-kb deletion analysis identified one copy of a five-bp deletion in exon 16 of the *GALC* gene. A second mutation was not identified. After the affected individual passed away, samples from the parents were sent to EGL for testing. The individual’s mother was found to carry the five-bp deletion. Array-CGH deletion/duplication testing of the individual’s father detected a 6.9-kb deletion of exon 8 (see figure 1).

### Case 2

**Figure 2:**
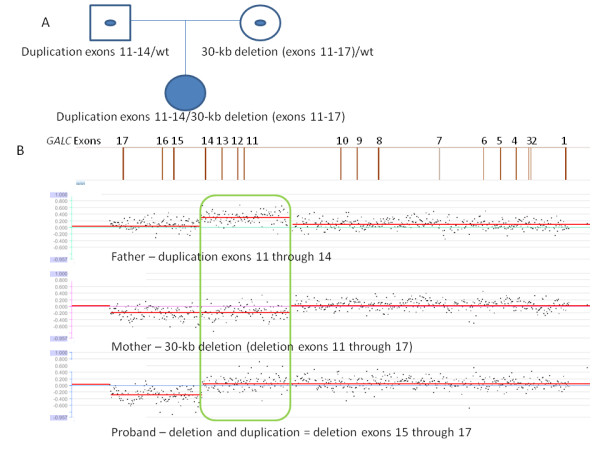
**Case 2. A. Pedigree of the proband and parents.** A filled symbol indicates the affected individual; symbols with a dot in the middle indicate carriers. *GALC* genotypes are given for each individual under the respective symbol. Sequence analysis with 30-kb deletion analysis identified one copy of the 30-kb deletion in the proband and her mother. A second mutation was not identified. Array CGH analysis was performed on the proband’s father to identify the second mutation, which was confirmed in the proband. **B.***GALC* array CGH results for the father (top), mother (middle), and proband (bottom). A diagram of the *GALC* gene is given above the results with exon numbers indicated. The father carries a duplication of exons 11 through 14, while the mother carries the 30-kb deletion of exons 11 through 17. The combination of the duplication and deletion in the proband yields a neutral copy number for exons 11 through 14 (boxed region), since she has two copies of those exons (both from her father and none from her mother), while exons 15 through 17 are deleted (present in only one copy from her father).

Case 2 was a one-year-old Caucasian female reported to have deficient GALC enzyme activity by blood assay and by fibroblast assay, and clinical features consistent with Krabbe disease. Sequence analysis with 30-kb deletion analysis identified one copy of the 30-kb deletion in this individual. A second mutation was not identified. Testing of the parents indicated that the child’s mother was the carrier of the 30-kb deletion. Array-CGH deletion/duplication analysis of the individual’s father revealed a novel 11-kb duplication encompassing exons 11 through 14. Subsequent deletion/duplication testing of the affected individual revealed a complex copy number pattern in the *GALC* gene resulting from the overlapping combination of the 30-kb deletion, which extends from exons 11 through the end of the gene, and a duplication of exons 11 through 14 (see figure 2). In this individual, the combination of a deletion and a duplication resulted in an array result with normal copy number for exons 11 through 14, while exons 15 through the end of the gene were deleted. In the absence of parental testing, this combined set of copy number changes would have been difficult to detect and interpret. This individual was confirmed to be the same individual reported in [10] and is the only report of a large duplication in the *GALC* gene to date.

### Case 3

**Figure 3:**
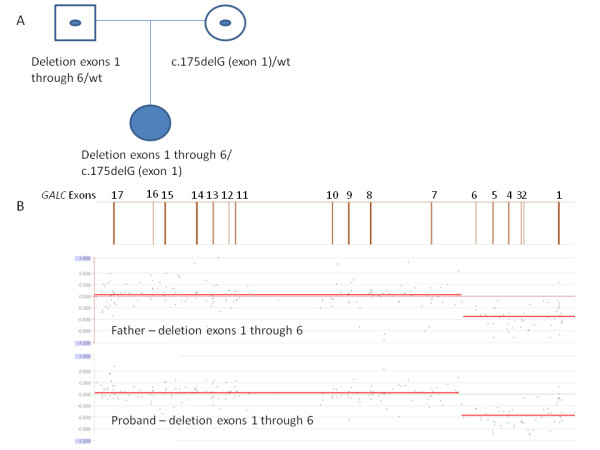
**Case 3. A. Pedigree of the proband and parents.** A filled symbol indicates the affected individual; symbols with a dot in the middle indicate carriers. *GALC* genotypes are given for each individual under the respective symbol. Sequence analysis performed in another laboratory identified an apparently homozygous one nucleotide deletion mutation in the proband. One copy of the mutation was identified in the proband’s mother, but her father was negative. Array CGH analysis was performed on the proband’s father to identify a suspected deletion, which was confirmed in the proband. **B.***GALC* array CGH results for the father (top) and proband (bottom). A diagram of the *GALC* gene is given above the results with exon numbers indicated. Both the father and the proband have one copy of a deletion of exons 1 through 6. The deletion inherited from her father made the mutation in exon 1 inherited from her mother appear homozygous in the proband by sequence analysis.

Case 3 was a 17-month-old East Indian female. Testing in another laboratory indicated deficient GALC activity and a homozygous single base pair deletion in exon 1 of the *GALC* gene. Testing of this individual’s parents indicated that the mother was a carrier of the single base pair deletion, but testing for the mutation in the father was negative. Samples were submitted to EGL for array-CGH deletion/duplication testing for the affected individual and her father. This testing revealed a deletion of exons 1 through 6 in both individuals (see figure 3). The deletion that the affected individual inherited from her father caused the mutation inherited from her mother to appear homozygous by sequence analysis.

## Discussion

Array CGH is currently being used successfully in many molecular cytogenetic laboratories to detect gross alterations in the human genome. Most cytogenetic arrays, however, are not designed to detect small (intragenic) deletions and duplications. The development of a clinical test for detecting intragenic copy number changes has the potential to raise the mutation detection rate for tested diseases, thereby improving molecular diagnosis of affected individuals. Gene-targeted array CGH offers a powerful alternative to the current methods used for detecting these mutations.

We have adapted array CGH technology and successfully shown that it can detect intragenic deletions and duplications in a large set of genes, including *GALC*, using a single array [11]. A study by Aradhya et al. demonstrated that gene-targeted array CGH was able to identify partial or whole gene deletions and duplications in approximately 5% of a clinical cohort sent to a diagnostic laboratory for testing [12]. When broken down by mode of inheritance, the positive rate approximately 5% for autosomal dominant genes, approximately 10% for autosomal recessive genes, and approximately 3.5% for X-linked genes. They conclude that intragenic copy number mutations are more prevalent that previously suspected in Mendelian disorders and should be part of their routine diagnostic workup. In addition, a study by Wang et al. reported a 3% positive rate when using gene targeted array CGH to identify deletions and duplication in a mitochondrial and metabolic patient cohort, and conclude that gene targeted array CGH is useful as a complementary diagnostic test for gene sequence analysis [13].

The case reports presented here demonstrate the utility of gene-targeted array CGH in clinical molecular diagnostics to improve and clarify mutation detection, as well as to identify copy number mutations that otherwise would have been missed by conventional analysis. As case 3 illustrates, apparently homozygous mutations in affected individuals should be verified by testing parents for the mutation and/or performing deletion/duplication analysis on the affected individual to rule out the presence of one copy of the mutation on one allele and a deletion on the other allele. Of the 11 individuals in this study with apparently homozygous mutations identified by sequence analysis with 30-kb deletion analysis (seven point mutations and four 30-kb deletions), parental samples were submitted on only three; in all three cases, the parents were shown to each carry one copy of the now verified homozygous mutation identified in the child. Without parental testing on the other eight cases, it is not possible to rule out the presence of a deletion, possibly leading to an underrepresentation of the frequency of novel deletions in the *GALC* gene.

In addition, the novel 11-kb duplication of exons 11-14 in case 2 would not have been detected by the use of traditional methodologies. This duplication normalizes the copy number in the proband from exons 11-14, in spite of the fact that the proband carries the common 30-kb deletion on the other allele. The detection of a deletion and duplication *in trans* in the same gene with partially overlapping exons clearly proved the power of aCGH to detect gene-targeted deletions and duplications. The better able we are to detect mutations, the more options there will be for molecular prenatal testing and carrier testing in family members.

## Conclusions

**Figure 4:**
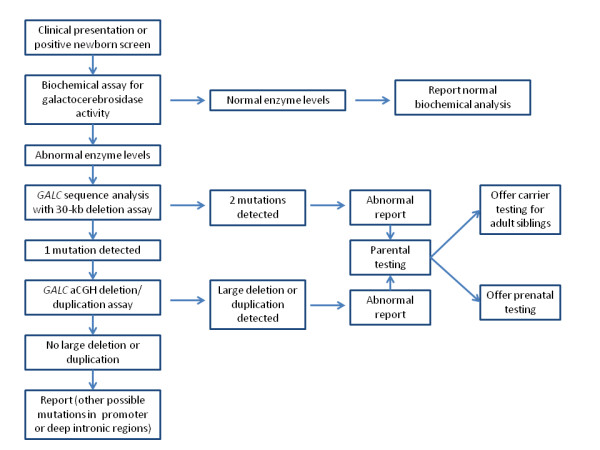
Algorithm for testing for Krabbe disease in affected individuals.

An algorithm for molecular testing for Krabbe disease is given in figure 4. First, genetic counselling and molecular testing for Krabbe disease can be offered to individuals with clinical symptoms, low levels of GALC enzyme, or positive newborn screen results. Initial testing should begin with *GALC* gene sequence analysis, along with allele-specific PCR for the 30-kb deletion, as these methods have the highest detection rate and are cost effective. If these methods do not identify two known mutations, *GALC* array CGH deletion/duplication analysis should be offered. If two mutations are identified, prenatal testing can be offered after parental testing to confirm that the two mutations are *in trans*. Since sequence analysis alone cannot distinguish a homozygous mutation from a sequence mutation that lies in *trans* with a deletion in the same gene, mutation-specific testing of both parents of individuals with an apparently homozygous mutation is especially important. Parental testing should be followed by deletion/duplication testing in those cases in which one parent tests negative for the mutation. Once two mutations have been identified and confirmed in parental samples, testing by known mutation analysis would also be possible for other affected family members or as carrier testing in adult family members.

This study of 33 Krabbe cases reveals that array CGH deletion/duplication analysis of the *GALC* gene increased the rate of detection of two mutations from 84.8%, for sequence analysis with 30-kb deletion analysis, up to 93.9%, and enabled us to uncover three previously unreported copy number mutations. In addition to the 33 cases presented here, there were also three other cases for which sequence analysis with 30-kb deletion analysis failed to identify two known mutations, but for which reflex deletion/duplication testing was not ordered. In one case, only one mutation was detected though sequence analysis, whereas in the other two cases, one known mutation and one variant of unknown clinical significance were detected through sequence analysis. In all three cases, deletion/duplication analysis and parental testing were recommended in the hope that a second mutation could be identified or the significance of the variants might be clarified; none of this testing, however, has been ordered at EGL to date. Ultimately, further *GALC* array CGH deletion/duplication testing in clinical laboratories will likely refine these statistics and identify other novel mutations.

## Methods

### Sequencing

Oligonucleotide primers were designed to amplify the 17 coding exons of the *GALC* gene in 16 fragments. (Oligonucleotide sequences are available from the authors upon request.) PCR products were analyzed on a 2% agarose gel, after which the remainder of the PCR product was purified using a Millipore Ultrafiltration PCR purification kit (Millipore, Billerica, MA). Sequencing reactions were prepared in a 10-ul reaction volume using the BDv3.1® sequencing kit (Applied Biosystems, Foster City, CA). Each PCR product was sequenced in both directions according to ABI recommendations using universal M13 sequencing primers. Sequenced PCR products were purified using Sephadex® cleanup plates by Edge Biosystems (Gaithersburg, MD) according to the manufacturer’s recommendation. Samples were heat-denatured for 5 minutes and loaded onto an ABI 3730xl sequencer. Sequence data were analyzed using Mutation Surveyor® v3.1 (Softgenetics, PA) and SeqScape® (Applied Biosystems, Foster City, CA).

### Allele-specific PCR

The allele-specific PCR assay for the 30-kb *GALC* deletion was designed by Rafi et al., 1995. Three primers, one sense and two antisense, were used to amplify the wild-type and mutant alleles. The PCR products were analyzed on a 2% agarose gel. A wild-type allele yields a 615 bp PCR product whereas a mutant allele yields a 320 bp PCR product.

### Array CGH

#### Array design

The targeted gene high-resolution oligonucleotide CGH array was custom designed on Oxford Gene Technologies (OGT) 180 K platform to detect deletions and duplications in 175 genes associated with various genetic disorders. Long oligonucleotides (~45–60 mer) were used to design the array, with repeat sequence masking implemented to ensure greater sensitivity and specificity. The *GALC* gene was covered by 431 probes with 116 probes covering the 17 exons at an average spacing of 15 bp between probes. The intronic region was covered by 315 probes at an average spacing of 25 bp between probes. Use of intronic oligonucleotide probes allows us to detect dosage changes within the entire genomic region of the gene and determine the approximate breakpoints.

#### Experimental set up

DNA extraction was performed on patient DNA using a Gentra Puregene DNA extraction kit according to the manufacturer’s instructions. Male and female wild-type control DNA was obtained from Promega, Inc. Each patient and reference DNA sample was sonicated, such that the DNA fragment size was between 200-5,000 bases and verified on a 1% agarose gel. Patient and reference DNA samples were labeled using Klenow enzyme (NEB) and Cy3 or Cy5 9mer wobble primers (TriLink Technologies), respectively. After labeling, each sample was purified by isopropanol precipitation and reconstituted in ultra-pure water. We combined 4 ug each of labeled patient and reference DNA, and the products were desiccated in a vacufuge (Savant DNA 120), then resuspended in appropriate hybridization buffer along with Cy3 and Cy5 control CPK6 50mer oligonucleotides. This mixture was hybridized to a NimbleGen targeted gene CGH array for 16-20 hours at 42 °C in a Maui Hybridization system (BioMicro Systems). The array has 389,587 unique sequence probes with an average spacing of 10-bp within coding regions and 25-bp within intronic regions, allowing for detection of copy number changes and breakpoints as small as 100-bp within the entire coding region. Arrays were then washed according to the manufacturer’s recommendation and immediately scanned on a GenePix 4000 scanner (Molecular Devices).

After scanning, data were extracted from images, and within-array normalization was accomplished using manufacturer-provided software (NimbleScan). Normalized log(2) ratio data were analyzed using two different analysis programs: SegMNT and DNA copy NimbleScan (NimbleGen Systems, Inc.). Both software programs report breakpoints for predicted deletions or duplications in the patient or test sample relative to the reference and also display results in a bar graph where the y-axis indicates gain or loss of material (1 = gain, 0 = normal, -1 = loss), while the x-axis indicates the position of each feature on the chromosome.

Data files (.gff) generated from different averaging windows using NimbleScan software were parsed using a custom program (Nimkit) that was developed in-house. Nimkit enables the laboratory to select and analyze only the gene of interest. Nimkit generates a gene-specific report summarizing breakpoints detected in the gene of interest, the respective log(2) ratios, and the exons present at each region. All other regions are masked and not analyzed by Nimkit, preventing genetic analysis of genes for which clinical testing was not requested, in compliance with HIPAA requirements.

Array quality was assessed by control resequencing oligonucleotides on each array that correspond to synthetic sequences designed to have no cross-hybridization potential to any known sequence. This sequence was designed to have three distinct sequencing domains with different characteristics: A, B, and C domains. Resequencing was performed on both the forward and reverse strands, so that the resequencing report has six different scores for the Cy3 channel and six distinct scores for the Cy5 channel: A-forward and A-reverse, B-forward and B-reverse, C-forward and C-reverse.

The “A” domain contained long runs of G nucleotides that can be difficult to synthesize. The “B” domain contained a large perfect hairpin sequence. The “C” domain contained a straightforward domain that should always resequence. Failure of domain “C” indicated a catastrophic failure. Control DNA was spiked into each experiment for both CGH and resequencing arrays. A score from 0-100% was obtained that indicated the sequence fidelity and correlated well with the overall performance of a microarray experiment [14].

## Abbreviations

CGH, Comparative genomic hybridization; GALC, Galactocerebrosidase; MLPA, Multiplex ligation-dependent probe amplification; DOVAM-S, Detection of virtually all mutations-SSCP; SCAIP, Single condition amplification/internal primer sequencing; EGL, Emory Genetics Laboratory.

## Competing interests

The authors declare no competing interests.

## Authors’ contributions

AKT participated in data analysis and drafted the manuscript. ELHC participated in performing the sequence analysis and array CGH analysis. PKD collaborated on the case of the large *GALC* gene duplication. MH conceived of the study, and participated in its design and coordination and helped to draft the manuscript. All authors read and approved the final manuscript.
